# Protein Composition of *Mycobacterium smegmatis* Differs Significantly Between Active Cells and Dormant Cells With Ovoid Morphology

**DOI:** 10.3389/fmicb.2018.02083

**Published:** 2018-09-04

**Authors:** Kseniya Trutneva, Margarita Shleeva, Vadim Nikitushkin, Galina Demina, Arseny Kaprelyants

**Affiliations:** A.N. Bach Institute of Biochemistry, Federal Research Centre ‘Fundamentals of Biotechnology’ of the Russian Academy of Sciences, Moscow, Russia

**Keywords:** dormant mycobacteria, *Mycobacterium smegmatis*, 2D electrophoresis, proteomic profile, dormancy models

## Abstract

Mycobacteria are able to form dormant cells, which survive for a long time without multiplication. The molecular mechanisms behind prolonged survival of dormant cells are not fully described. In particular, little information is known on biochemical processes which might take place in cells under dormancy. To gain insight into this problem, *Mycobacterium smegmatis* cells in deep dormant state were obtained after gradual acidification of the growth medium in prolonged stationary phase followed by 1 month of storage at room temperature. Such cells were characterized by low metabolic activity, including respiration, resistance to antibiotics, and altered morphology. The protein composition of cytoplasm and membrane fractions obtained from active and dormant cells were compared by 2D electrophoresis. Almost half of the proteins found in the proteome of dormant cells were absent in that of active cells. This result differs significantly from published results obtained in other studies employing different models of mycobacterium dormancy. This discrepancy could be explained by a deeper dormancy developed in the present model. A feature of a “dormant proteome” is high representation of enzymes involved in glycolysis and defense systems that inactivate or detoxify reactive oxygen and nitrogen species, aldehydes, and oxidized lipids. Dormant mycobacteria are enriched by degradative enzymes, which could eliminate damaged molecules, or the products of such degradation could be reutilized by the cell during prolonged storage. We suggest that some enzymes in dormant cells are inactive, having been used upon transition to the dormant state, or proteins stored in dormant cells for further cell reactivation. At the same time, some proteins could be functional and play roles in maintenance of cell metabolism, albeit at a very slow rate. This study provides a clue as to which biochemical processes could be active under dormancy to ensure long-term viability of dormant mycobacteria.

## Introduction

*Mycobacterium tuberculosis* (*Mtb*) is a most successful pathogen, which can persist in the human host for a long period, forming non-dividing dormant cells and causing asymptotic latent tuberculosis (LTB). Despite intensive studies aiming to elucidate the molecular mechanisms behind the formation of dormant *Mtb* and the prolonged survival of mycobacteria, our knowledge is not complete. We have limited information on the biochemical processes which might occur in cells in the dormant state. The most known studies in this area have been conducted using anaerobic Wayne model for *Mtb* (Wayne, 1994). To mimic *Mtb* dormancy *in vitro*, the rapidly growing nonpathogenic microorganism *Mycobacterium smegmatis* (*Msm*) is widely used. For this model, cell transition into the dormant state upon slow oxygen depletion (Cunningham and Spreadbury, 1998; Dick et al., 1998), sub-lethal concentration of vitamin C (Mishra and Sarkar, 2015) and as a result of adaptation to prolonged stationary phase (Shleeva et al., 2004) was shown.

However, non-replicative cells obtained in Wayne and other *in vitro Mtb* models (Loebel et al., 1933; Wayne, 1994; Rustad et al., 2008) reveal fully culturable and metabolically active bacteria. At the same time, dormant cells in latently infected individuals are characterized by non-culturability (transient inability to grow on non-selective solid media; Khomenko and Golyshevskaya, 1984; Dhillon et al., 2004; Chao and Rubin, 2010). Moreover, by definition, non-active dormant cells should be resistant to antibiotics, which is not the case for cells in the Wayne model nor other models (Salina et al., 2014). Only the Cornell *in vivo* model for LTB could be considered to most adequately mimic the real situation. However, due to very low numbers of dormant cells in the Cornell model, this model is difficult to explore for biochemical characterization of those cells. We suggested two *in vitro* dormancy models of *Mtb* and *Msm* that meet such important criteria as low metabolic activity and resistance to antibiotics (Kudykina et al., 2011; Shleeva et al., 2011) and non-culturability (Salina et al., 2014). For this study we explore one of these models (formation in significant amounts of dormant *Msm* cells with distinct morphology under gradual external acidification (Kudykina et al., 2011). The main aim of our study was to conduct comparative 2D proteome analysis of active and dormant *Msm* cells in order to uncover a set of stable and enzymatically active proteins under prolonged storage. We will also use this knowledge to make suggestions about biochemical processes which might take place under development and maintenance of *Msm* cells during prolonged dormancy.

## Materials and Methods

### Bacterial Strains, Growth Media, and Culture Conditions

*Mycobacterium smegmatis* mc^2^ 155 was initially grown for 24 h in nutrient broth (“Himedia”) (NBE) in the presence of 0.05% Tween-80 at 37°C under agitation (220 rpm). The resulting culture served as an inoculum that was added to 250 ml of modified Sauton medium (pH 7.0) at a concentration of 10^5^–10^6^ cells/ml, containing (per liter): 0.5 g KH_2_PO_4_; 1.4 g MgSO_4_⋅7H_2_O; 4 g L-asparagine; 60 ml glycerol; 0.05 g ferric ammonium citrate; 2 g sodium citrate, and 0.1 ml 1% ZnSO_4_⋅7H_2_O, supplemented with 0.05% Tween-80. The modification of Sauton medium included reduction of the initial pH to 6.0–6.2 (no addition of NaOH) (Kudykina et al., 2011). Cultures were incubated in modified Sauton medium at 37°Ñ with shaking for 10–20 days, and pH values were periodically measured. When *Msm* cultures were in the post-stationary phase and had reached pH 6.0–6.2 (after 13–15 days), cultures were transferred to plastic-capped tubes (50 ml) and maintained under static conditions without agitation at room temperature for up to 30 days post inoculation.

### Microscopy

Cell suspensions were examined using the phase-contrast and epifluorescence microscopy after staining with propidium iodide (PI) (3 mM) to detect injured cells. Phase-contrast epifluorescence microscopy was carried out on Nikon eclipse Ni-U microscope, magnification ×1,500. Epifluorescence microscopy was carried out in the “TRITC channel” (Ex = 540/25 nm; DM = 565 nm; BA = 605/55 nm). Photos were taken using Nikon DS Qi2 camera (Japan).

### Viability Evaluation by MPN

Most probable number (MPN) assays of *Msm* were performed in 48-well plastic plates (Corning) containing 1 ml Sauton medium diluted in NBE (1:1). Appropriate serial dilutions of *Msm* cells (100 μl) were added to each well. Plates were incubated at 37°C with agitation at 130 rpm for 7 days. Wells with visible bacterial growth were counted as positive, and MPN values were calculated using standard statistical methods (de Man, 1974).

### Viability Evaluation by CFU

Bacterial suspensions were serially diluted in fresh Sauton medium diluted in NBE (1:1), and three replicates of 100 μl samples from each dilution were spotted on NBE agar. Plates were incubated at 37°C for 4–5 days, and colony-forming unit (CFU) were counted. The lower limit of detection was 10 CFU/ml.

### Metabolic Activity Estimation

Cell metabolic activity was determined by incorporation of 1 μl of [5,6-^3^H]-uracil (1 μCi, 0.02 μmol) into cells culture (1 ml) and incubation for 4 h at 37°C with agitation and at room temperature without agitation. Cells culture (200 μl) were then harvested on glass microfiber GF/CTM filters (Whatman, United Kingdom) and washed with 3 ml 7% trichloroacetic acid followed by 3 ml absolute ethanol. Air-dried filters were placed in 5 ml of scintillation liquid (Ultima GoldTM, Perkin Elmer, United States), and the radioactivity incorporated was measured with a scintillation counter LS6500 (Beckman, United States).

### Respiratory Chain Activity

Endogenous respiratory chain activity was determined spectro photometrically by reduction of 2,6-dichlorophenolindophenol (DCPIP) (OD_600_) and by oxygen consumption polarographically. DCPIP is a synthetic electron acceptor which is reduced by active respiratory chain via menaquinone. Endogeneous DCPIP reductase was assayed by the decrease of OD600. The reaction mixture (4 ml) contained 0.2 μM 2,6-DCPIP, 0.6 μM menadione, and 400 μl of the cell suspension in phosphate buffer saline (PBS, pH 7.4).

### Antibiotic Sensitivity Testing

One milliliter of early stationary phase culture *Msm* (active bacteria) grown in Sauton medium (pH 7.0) for 2 days or 1 ml of the dormant cell culture were diluted in their own supernatants to 10^8^ cells/ml. Then cells were treated with 50 μg/ml of rifampicin or bedaquiline and kept at 37°C for 7 days without agitation. Cells were incubated with 100 μg/ml hygromycin for 1 day. The number of resistant cells was determined by the MPN assay (see above).

### Sample Preparation for 2D Electrophoresis

Active and dormant cells obtained in four biological replicates were pooled for 2D electrophoretic analysis. Bacteria were harvested by centrifugation at 7,000 rpm 15 min and washed 10 times with a buffer containing (per liter) 8 g NaCl, 0.2 g KCl, and 0.24 g Na_2_HPO_4_ (pH 7.4). The bacterial pellet was re-suspended in ice-cold 100 mM HEPES (4-(2-hydroxyethyl)-1-piperazineethanesulfonic acid) buffer (pH 8.0) containing complete protease inhibitor cocktail (Sigma, United States) and phenylmethanesulfonyl fluoride (PMSF) then lysed by using zirconium beads on a bead beater homogenizer (MP Biomedicals FastPrep-24) for 1 min, five times for active cells and 10 times for dormant cells. The bacterial lysate was centrifuged at 13,000 rpm for 15 min at 4°C. The supernatant was separated into membrane and cytosolic fractions using ultracentrifugation at 100,000 × *g* for 2 h. The membrane fraction was washed with HEPES buffer three times using ultracentrifugation. To isolate the membrane fraction, the zwitterionic detergent CHAPS (3-(3-cholamidopropyl) dimethylammonio-1-propanesulfonate hydrate (2%) (w/v) was used. After first extract on membranes were washed with HEPES three times, and a second extraction was performed using the strong anionic detergent sodium dodecyl sulfate (SDS) (2%) (w/v). The cytosolic fraction and two membrane extracts were precipitated using the ReadyPrep 2-D cleanup kit (BioRad, United States) for selective precipitation to remove ionic contaminants such as detergents, lipids, and phenolic compounds from protein samples. This kind of precipitation allows resuspension of the protein pellet in isoelectric focusing buffer contains 8 M urea, 2 M thiourea, 10 mM 1,4-dithiothreitol (DTT), 2 mM TCEP (Tris(2-carboxyethyl)-phosphine-hydrochloride), 1% (w/v) CHAPS, 1% (w/v) Triton X-100, 1% (w/v) amidosulfobetaine-14 (ASB), and 0.4% (v/v) ampholytes (pH 3–10).

### Two-Dimensional Electrophoresis

Isoelectric focusing was performed in a 5% acrylamide gel (30% (w/v) acrylamide/bisacrylamide, 8 M urea, 2% (v/v) ampholyte pH 3–10, and 4–6 (1:4), 1% (w/v) CHAPS, 1% (w/v) Triton X-100, 0.4% (w/v) ASB) using a 2.4-mm ID glass tubes in a Tube Cell (Model 175, BioRad, United States) until 3,700 V h were attained. After focusing, gels were extracted from the glass tubes and fixed in equilibration buffer 1 (0.375 M Tris-HCl, pH 6.8, 2 M urea, 20% (v/v) glycerol, 2% (w/v) SDS, 2% (w/v) DTT) and equilibration buffer 2 (0.375 M Tris-HCl, pH 6.8, 2 M urea, 20% (v/v) glycerol, 2% (w/v) SDS, and 0.01% (w/v) bromophenol blue) for 15 min each. Second-dimension separation was performed as described by O’Farrell (1975) in large format (20 cm × 20 cm), 1.5 mm thick 12% SDS-PAGE gels in standard Tris-glycine buffer in a PROTEAN II xi cell for vertical electrophoresis (BioRad, United States). The gels were stained by Coomassie CBBG-250 (Roti-Blue Carl Roth, Germany) followed by silver staining^1^.

The gel image was captured using Syngene G:BOX Gel & Blot Imaging Systems (Syngene, United Kingdom). Gel images stained by Coomassie were analyzed using TotalLab TL120 software to calculate spot density.

Each visible protein spot was excised manually from the gel and analyzed like separated sample using matrix-assisted laser desorption/ionization time-of-flight (MALDI-TOF) analysis. The MS/MS data obtained from MALDI-TOF were subjected to a Mascot Protein Database (MSDB) search to identify proteins. Proteins with the coverage less than 12% were not further considered. Protein functional roles for *Msm* were obtained from the Tuberculist database for corresponding orthologs in *Mtb*. Each sample for 2D analysis was performed in two technical replicates.

### Protein Identification by MALDI-TOF

All fractions excised from 2D electrophoresis slab gels were hydrolysed by trypsin digestion. The extracted tryptic peptides were analyzed by MALDI-TOF mass spectrometry as described previously, with some modifications. A sample (0.5 μl) was mixed with the same volume of 20% (v/v) acetonitrile solution containing 0.1% (v/v) trifluoroacetic acid and 20 mg/ml 2,5-dihydroxybenzoic acid and then air-dried. Mass spectra were obtained on a Reflex III MALDI-TOF mass spectrometer with a UV laser (336 nm) in positive-ion mode in the range of 500–8,000 Da. Calibration was performed in accordance with the known peaks of trypsin autolysis.

For MS/MS analysis, the mass spectra of fragments were recorded with a Bruker Ultraflex MALDI-TOF mass spectrometer in tandem mode (TOF–TOF) with detection of positive ions. The proteins were identified using Mascot software in Peptide Fingerprint mode (Matrix Science, Boston, MA, United States). The accuracy of the mass measurement MH+ was 0.01% (with a possibility of modifying cysteine by acrylamide and methionine oxidation).

### Metabolite Determination

#### Total Thiol Assay

Bacteria (∼80–100 mg) were pelleted by centrifugation, and the pellet was resuspended in a mixture (pH 7.4) of 20 mM HEPES containing 10 mM ethylenediaminetetraacetic acid (EDTA) with 50% (v/v) acetonitrile. The samples were heated to 60°C for 10 min to extract the cellular contents. Two milligrams of DTNB [5,5′-dithio-bis-(2-nitrobenzoic acid)] was dissolved in 3 ml HEPES–EDTA buffer (pH 7.4). The reaction mixture contained 100 μl of the sample, 100 μl of the DTNB solution, and 700 μl of the HEPES–EDTA buffer. Thiols react with DTNB to form TNB (5-thio-2-nitrobenzoic acid). The TNB formed was quantified at 412 nm. Three independent replicates were performed for each sample.

#### Cellular Concentrations of NADH and NAD+

Samples containing 100 mg wet cells were placed in Eppendorf tubes and centrifuged at 13,000 rpm for 3 min. After removal of the supernatant, the pellets were treated either with 300 μl 0.2 M HCl (NAD extraction) or 300 μl 0.2 M NaOH (NADH extraction). Specific dinucleotides were extracted by placing the samples in a thermostat at 75°C for 10 min. After incubation, the suspensions were cooled to 0°C and neutralized by adding equimolar amounts of either HCl or NaOH. After centrifugation, the dinucleotide-containing supernatants were collected and transferred to a new tube and used immediately.

Extracts were analyzed with the recycling assay of Bernofsky and Swan (1973). The assay reagents contained: 0.6 mM of 3-[4,5-dimethylthiazol-2-yl]-2,5-diphenyltetrazolium bromide, 3.7 mM of phenazine methosulfate, 3.4 mM of ethanol, 0.14 mM tricine (pH 8.0), 5.0 mM EDTA (pH 8.0), and 7 U/ml yeast alcohol dehydrogenase (ADH). The yeast ADH converts NAD+ to NADH, utilizing ethanol. For the assay the reduction of MTT at 570 nm in a Cary UV/Vis spectrophotometer was monitored. Three independent replicates were performed for each sample.

#### cAMP Determination

Cell culture (1 ml) at different stages of growth (24 h) and storage (1 month) were centrifuged at 13,000 *g* for 5 min. The cell pellets were treated with 1 ml 0.1 N HCl, then samples were heated at 95°C for 5 min and frozen immediately. The collected samples were disrupted by using a bead homogenizer FastPrep-24, bacterial debris were removed by centrifugation, and aliquots of the lysate were taken for estimation of cAMP. cAMP levels were measured by ELISA using rabbit cAMP antibody (1:5,000) (GenScript, United States) and cAMP-HRP (1:20,000) (cAMP-peroxidase conjugate, GenScript, United States). The results were registered at 450 nm with a Zenyth 3100 microplate reader (Anthos Labtec Instruments, Austria). Three independent replicates were performed for each sample.

#### ATP Determination

Cell culture (1 ml) were collected at two time points [active growth 24 hand dormancy (1 month of storage)] were centrifuged at 13,000 *g* for 5 min. The cell pellets were washed with PBS pH 7.5. Cells were destroyed in PBS using bead homogenizer. Cell debris were separated by centrifugation, 10-μl aliquots of supernatant were mixed with “ATP reagent” (100 μl) (Lumtek, Russia) and luminescence was measured by a chemiluminometer Lum-5773 (Disoft, Russia). ATP standards ranging from 0.1 to 10 nM were prepared fresh for each experiment. Three independent replicates were performed for each sample.

### Enzymatic Assays

#### Sample Preparation

Active and dormant cells (∼100 mg wet weight) were harvested by centrifugation and washed with PBS once followed by homogenization using zirconium beads in a FastPrep-24 bead beater homogenizer in 1 ml of ice-cold PBS (6 cycles, 25 s each). Debris were discarded by centrifugation at 15,000 *g* for 15 min. The cell extract was used for further enzymatic activity determination. Three independent replicates were performed for each sample in each assay.

#### Alcohol Dehydrogenase

Alcohol dehydrogenase specific activity was determined by measuring the rate of oxidation of 0.25 mM NADPH at 340 nm in 0.02 M KH_2_PO_4_/Na_2_HPO_4_ buffer (pH 7.3) and in the presence of 50 μM benzaldehyde and 0.2 ml of cell extract (Galamba et al., 2001).

#### Glycerol-3-Phosphate Dehydrogenase

Activity was quantitated by determining the phenazine methosulfate-coupled reduction of 2-(4,5-dimethyl-2thiazolyl)-3,5-diphenyl-2H-tetrazolium bromide. The reaction was measured spectrophotometrically at 570 nm in the presence of 1% (w/v) n-octyl-β-D-glucoside. Each cuvette contained 50 mM Tris-HCl (pH 7.4) buffer, 75 mM sodium chloride, 0.01 M sodium cyanide, 0.5 mM MTT, 0.2 mM PMS, and 0.2 ml of cell extract. The reaction was initiated by the addition of 20 mM D,L-glycerol-3-phosphate (Yeh et al., 2005).

#### Glycerol Kinase

The rate of the reaction was measured in a coupled system with pyruvate kinase and lactate dehydrogenase. One unit results in the oxidation of 1 μmol of NADH at room temperature and pH 8.9. The reaction was measured spectrophotometrically at 340 nm in a cuvette, containing carbonate-glycine buffer (0.3 mM glycine, containing 30 mM potassium carbonate, pH 8.9), 2 mM ATP, 0.3 mM NAD^+^, 0.5 mM PEP, 6.5 mM MgSO_4_, 6 mM reduced glutathione, 3 mM glycerol, 3.5 U/ml lactate dehydrogenase, and 1.6 U/ml pyruvate kinase. The reaction was started by the addition of 0.2 ml of the cell extract.

#### Glyceraldehyde-3-Phosphate Dehydrogenase

The reaction velocity was measured as an increase in absorption at 340 nm resulting from the reduction of NAD in a cuvette, containing 10 mM sodium phosphate buffer (with the addition of 20 mM sodium arsenate), pH 8.5, 0.25 mM NAD^+^, 3 mM DTT and 0.25 μM D-glyceraldehyde-3-phosphate. The reaction was started by the addition of 0.2 ml of the cell extract.

#### Phosphoglycerate kinase

The enzymatic activities were assayed in the reverse direction, from 3-phosphoglycerate to 1,3-bisphosphoglycerate. The reaction mixture was composed of 80 mM triethanolamine buffer (pH 7.6), 8.0 mM MgSO_4_, 0.25 mM NADH, 2.4 mM ATP, 12 mM 3-phosphoglycerate, 50 μg/ml glyceraldehyde-3-phosphate dehydrogenase, and 0.2 ml cell extract. The reaction was measured by OD at 366 nm and 25°C. (Krietsch and Bucher, 1970).

#### Pyruvate Kinase

The velocity of the reaction was determined in a lactate dehydrogenase coupled assay by measuring the decrease in absorbance at 340 nm resulting from the oxidation of NADH. The reaction was measured spectrophotometrically in a cuvette containing 45 mM imidazole buffer, containing 0.1 M potassium chloride and 0.05 M MgSO_4_ (pH 7.6), 1,5 mM ADP, 0.22 mM NADH, 1.5 mM phosphoenolpyruvate, and 5 U/ml lactate dehydrogenase. The reaction was started by the addition of 0.2 ml of the cell extract.

#### Pyruvate Reductase

The reaction rate was determined from the decrease in absorbance at 340 nm resulting from oxidation of NADH. The reaction mixture contained 0.2 M Tris-HCl (pH 7.3), 0.2 mM NADH, 1 mM sodium pyruvate, and 0.2 ml of cell extract.

#### Lactate Dehydrogenase (Quinone-Dependent)

The reaction mixture contained 100 mM phosphate buffer (pH 7.5), 50 μM DCPIP, 20 mM L-lactate, 0.2 mM NAD, and 0.2 ml of cell extract. The reaction was measured spectrophotometrically at 340 nm (Molinary and Lara, 1958; Billig et al., 2017).

#### Isocitrate Lyase

The reaction mixture contained 30 mM imidazole (pH 6.8), 5 mM MgCl_2_, 1 mM EDTA, 4 mM phenylhydrazine and 1 mM D,L-isocitrate, and 0.2 ml of cell extract. The reaction was measured spectrophotometrically at 340 nm.

#### NADH Oxidase

The reaction rate was determined from the decrease in absorbance at 340 nm resulting from oxidation of NADH. The reaction mixture contained 0.2M Tris-HCl (pH 7.3), 0.2 mM NADH, and 0.2 ml of cell extract.

## Results

### Formation of Dormant *Mycobacterium smegmatis* Cells and Their Characterization

A population of dormant *Msm* cells in prolonged stationary phase was obtained by gradual acidification of the medium according to a published protocol (Kudykina et al., 2011). Dormant ovoid cells were kept in plastic-capped tubes in the dark at room temperature for an additional 1 month. The estimated viability of thusly stored dormant cells by CFU was unchanged/stable (∼1.5–5.0 × 10^9^ cells/ml in different experiments). The MPN assay revealed a viable cell number close to the CFU, reflecting almost full culturability of these dormant cells after this storage period.

**FIGURE 1:**
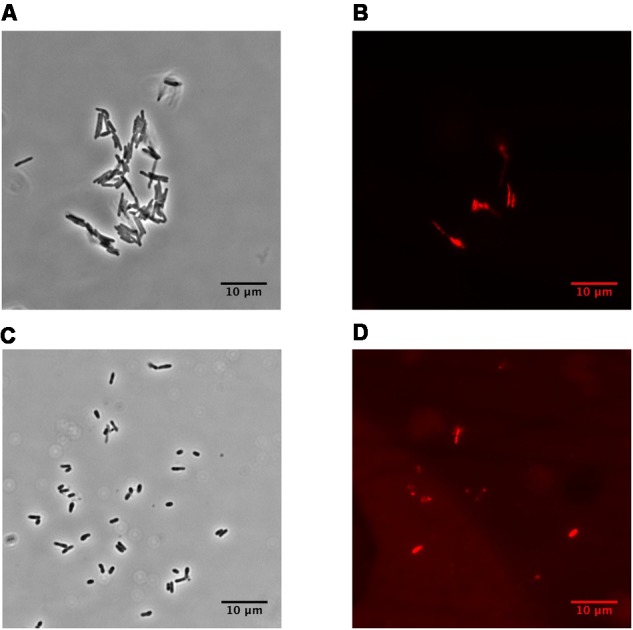
Phase-contrast and fluorescence microscopy of *M. smegmatis* cells (magnification ×1,500). **(A,B)** active, early stationary phase cells; **(C,D)** dormant cells after 1 month storage at room temperature. **(A,C)** phase contrast. **(B,D)** cells stained by propidium iodide to visualized dead cells. The bar below of each photo corresponds to 10 μm.

Dormant cells were characterized by fluorescent microscopy after staining with PI, which uncovers dead cells with a compromised cytoplasmic membrane (**Figure 1**). This approach revealed ∼60% intact cells in the population. Such cells appeared contrast, small and ovoid in comparison to the rod-shaped cells typical of multiplying bacteria (**Table 1**). Dormant cells were metabolically inactive, as indicated by a very low level of 5,6-^3^H uracil and low respiratory chain activity measured in either whole cells (reduction of electron acceptor DCPIP and oxygen consumption; **Table 1**) or in cell-free fraction (NADH oxidation; **Table 2**). We also checked intercellular concentration of ATP and cAMP – the molecules which accompany transition and exit from dormancy (Shleeva et al., 2011, 2013). Found detectable amounts of ATP and cAMP (**Table 1**) in dormant *Msm* cells favors the maintenance of some but low level of metabolism under dormancy. Dormant cells were significantly less sensitive to inhibition of RNA (rifampicin) and protein (hygromycin) synthesis or H^+^-ATPase (bedaquiline) (**Table 1**). The above mentioned properties of dormant cells reflect their more deep dormancy in comparison with cells obtained in published models, e.g., Wayne model. The obtained dormant cells were used for characterization of protein composition in comparison with that of active, multiplying cells.

**Table 1:** Some properties of active and dormant *M. smegmatis* cells.

Characteristic	Active cells	Dormant cells
Cell size(length/width, μm)	3.43 ± 1.05/0.61 ± 0.06	1.42 ± 0.35/0,55 ± 0.08
H^3^-Uracil inc. rate (CPM/mg wet cell weight)		
at 37° C	21,383 ± 3401	646 ± 13
at 25°C	6,036 ± 793	30 ± 5
Respiratory activity DCPI		
Preductase activity(OD_600_/ min^-1^ mg^-1^)	0.18 ± 0.01	0.01 ± 0.005
Oxygen consumption(nmol O_2_min^-1^ mg^-1^)	20 ± 4.5	2.5 ± 0.075
ATP (pmol × mg wetcell weight^-1^)	82 ± 13	10 ± 2
cAMP (pmol × mg wetcell weight^-1^)	116 ± 16	28 ± 6
Antibiotic resistence(%*)		
Rifampicin	0.02 ± 0.007	62.5 ± 17.8
Hygromycin	0.0001 ± 0.00005	10 ± 2
Bedaquiline	1.4 ± 0.5	30.8 ± 9.2

*^∗^The percent of cell population resistant to treatment with hygromycin (100 μg/ml) rifampicin (50 μg/ml) and bedaquiline (50 μg/ml) was determined from the ratios of MPN values.*

**Table 2:** Enzymatic activities and metabolite content in active and dormant *M. smegmatis* cells.

Enzymatic activities	Active	Dormant
(μmol/min/mg)		
1 Glycerolkinase	665 ± 49	78 ± 49
2 Glycerol-3-phosphate dehydrogenase	2,550 ± 190	93 ± 4
3 3-Phosphoglycerate-kinase	618 ± 46	241 ± 17
4 Glyceraldehyde-3-P-dehydrogenase	322 ± 15	108 ± 9
5 Pyruvate kinase	655 ± 23	395 ± 32
6 Lactate dehydrogenase (quinone-dependent)	350 ± 20	270 ± 30
7 Pyruvate reductase	0	55 ± 4
8 Lactate dehydrogenase	0	0
9 Alcoholdehydrogenase	200 ± 130	80 ± 40
10 Isocitrate lyase	68 ± 33	0
11 NADH oxidase (OD_340_)	221 ± 40	8 ± 3
**Metabolites**		
12 Total thiols (μmol/mg)	350 ± 160	820 ± 150
13 NAD (nmol/mg)	0.038 ± 0.012	0.0037 ± 0.001
14 NADH (nmol/mg)	0.153 ± 0.017	0.0137 ± 0.002
15 Ratio NADH/NAD	4.03	3.7

### Comparative Analysis of Proteomic Profiles

Two-dimensional electrophoresis was conducted separately for the membrane and cytosol fractions of active and dormant *Msm* for better protein separation and identification. In each experiment the protein amount used for the first dimension was identical for both types of cells, although the total amount of protein isolated from active cells was double the amount isolated from the same number of dormant cells. Typical results of 2D electrophoresis for the two protein fractions after Coomassie staining followed by silver staining are shown (2D photo; **Supplementary Figure S1**). Each protein spot was excised and identified by peptide mass fingerprinting using MALDI-TOF and MASCOT.

**FIGURE 2:**
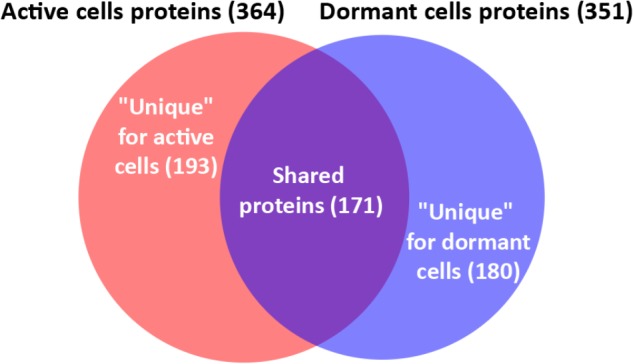
Venn diagram showing the overlap between active and dormant cells proteomic profile.

**FIGURE 3:**
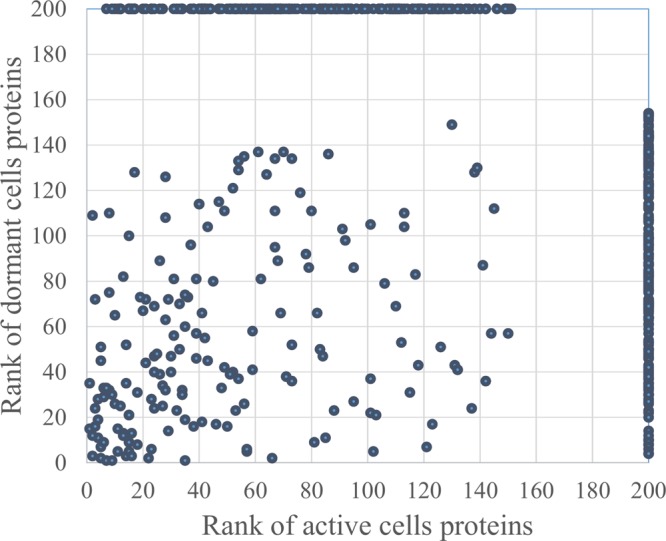
Proteins distribution according to their representation in the proteome of active and dormant *M. smegmatis*. Proteins were ranged according to their spot density from highest (1) to lowest (154 for active and 151 for dormant cells) representation in the proteome. Proteins were virtually absent in the proteome marked as “200” (**Supplementary Table S1**). Proteins within the area outlined by *dotted lines* were considered as similarly represented in both proteomes.

In total, 726 spots in different fractions of active and dormant bacteria were analyzed, and 586 proteins were identified as annotated in the “Smegmalist” database^2^. Each spot contained from 1 to 4 different proteins (on average, 1.2 proteins) in one spot. Among those, 364 and 351 proteins were identified in the active and dormant mycobacteria profiles, respectively (**Supplementary Table S1**). Only 170 proteins (∼50% in the proteome) were found to be identical (according to the accession number) for each bacterial type (**Figure 2**), which suggests a significant difference in protein expression profiles between active and dormant cells. Even among “common” proteins, the abundance of a particular protein in the whole proteome varied significantly (**Figure 3**). According to **Figure 3**, only 10% of identified proteins were represented similarly in both samples, indicating substantial changes in the metabolic processes under transition to dormancy. Approximately 50% of proteins in the “dormant proteome” were not represented in the “active proteome.” This protein cohort will be mentioned further as “unique” proteins (**Supplementary Table S2**). The term “unique” has an operational meaning for the present experimental conditions.

**FIGURE 4:**
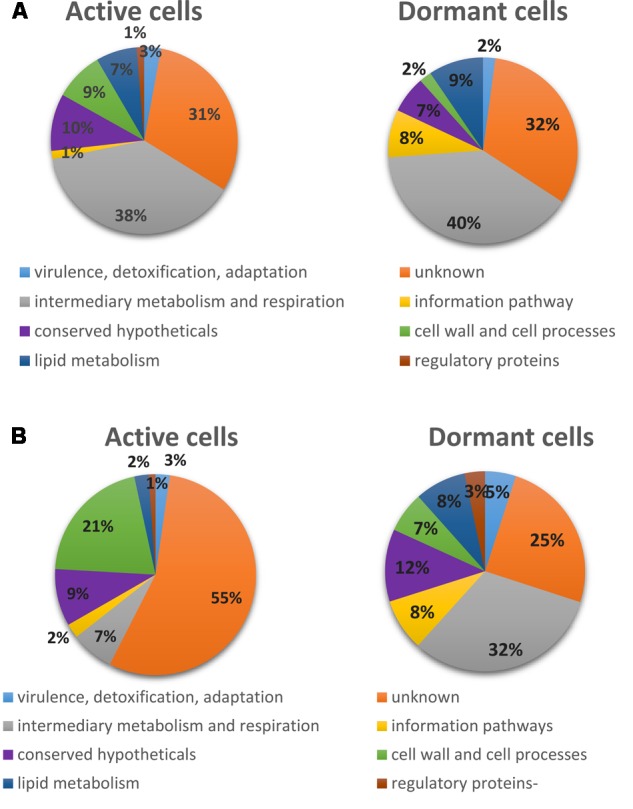
The identified proteins distribution in active and dormant *M. smegmatis* cells based on functional categories. Functional categories for *Mtb* orthologs in TubercuList was used for distribution of *M. smegmatis* proteins. **(A)** Cytosol fraction proteins. **(B)** Membrane fraction proteins (CHAPS and SDS extracts).

The protein distribution according to functional categories revealed a similar distribution between dormant and active cells in the cytosol fraction, in contrast to the protein distribution in the membrane fraction (CHAPS and SDS extracts) (**Figure 4**). In general, the “dormant proteome” contains fewer proteins in the category “cell wall processes” for both fractions in comparison with the “active proteome.” At the same time, the “dormant proteome” is enriched with proteins in the category “information processes.” Such changes evidently reflect suppression of metabolic processes involved in cell wall turnover in dormant cells and activation of regulatory mechanisms specific to the transition to dormancy.

Identified proteins were ranked by representation in the whole proteome, on the basis of spot density, for both active and dormant cells proteomes (**Supplementary Table S1**). This analysis makes it possible to avoid the difference in total protein concentration between active and dormant cells mentioned above and allows a comparison of individual protein expression between cell types. Similar approach has been used previously for comparative transcriptome analysis of active and dormant *M. tuberculosis* cells when the total amount of mRNA differs significantly between two types of cells (Salina et al., 2009). By using this algorithm, we analyzed the proteins with the aim of determining which metabolic pathways and enzyme activities might occur in dormant, morphologically altered *Msm* cells.

### Glycolysis

The conversion of glucose to pyruvate is accomplished by nine enzymes, eight of which were found in the “dormant proteome,” while phosphoglucomutase (MSMEG_2136) and phosphofructokinase (MSMEG_2366) were neither found in the active nor in the dormant proteome. Three enzymes were found in the “dormant proteome” only: pyruvate kinase 3 (MSMEG_3227), glucose-phosphate isomerase (MSMEG_5541), and fructose-bisphosphate aldolase (MSMEG_0752) (“unique” proteins; **Supplementary Table S2**). In order to verify activity of these enzymes biochemically, we checked activities of some glycolytic enzymes in cytosol fraction after cells disruption. According to **Table 2** all measured enzymes were active in dormant cells, some of them were on comparable level with active cells and one enzyme (pyruvate reductase) was measurable in dormant cells only.

### Citrate Cycle

The dormant cell proteome contains 10 key enzymes of the citric acid cycle, one of which (citrate synthase MSMEG_5676) was a “unique” protein. Among the enzymes included in the glyoxylate shunt, malate synthase MSMEG_3640 was found in both active and dormant cells. However, isocitrate lyase (Icl; MSMEG_0911/MSMEG_3706) was neither represented in the active nor in the dormant cell proteome. Estimation of the activity of this enzyme *in vitro* demonstrates its absence in dormant cells in contrast to active cells (**Table 2**), which calls into question the significance of this shunt in dormant *Msm*.

Conversion of asparagine to aspartate via highly represented in “dormant proteome” asparaginase (MSMEG_3173 “unique”; **Supplementary Table S2**) followed by oxidation of aspartate by aspartate oxidase or the interconversion of aspartate and α-ketoglutarate to oxaloacetate and glutamate via aspartate transaminase (MSMEG_6286) could be an additional source of oxaloacetate.

### Transcriptional Regulation

Protein factors of transcriptional regulation were found in both types of cells (TetR MSMEG_0859; MoxR MSMEG_3147; NusG MSMEG_1345; and LacI MSMEG_3599). However, some of them were represented in the active cell proteome only (Gre A MSMEG_5263; Crp/Fnr family protein MSMEG_0539; PhoP MSMEG_5872; MSMEG_3264; **Supplementary Table S2**). In ovoid cells, transcription factors were found in the membrane fraction only (NusA MSMEG_2625; IclR family MSMEG_3335 AmtR MSMEG_4300). Several factors were “unique” to dormant cells (PadR MSMEG_6227, AmtR MSMEG_4300, NusA MSMEG_2625; IclR family MSMEG_3335; SigB MSMEG_2752) and its regulator MprA (MSMEG_5488) (“unique” proteins; **Supplementary Table S2**).

### Defense Mechanisms

The proteome of dormant cells contained a significant number of proteins that participate in cell defense against harmful effects. Among the enzymes involved in defense against oxygen stress, the following are highly expressed: superoxide dismutases (MSMEG_6427; MSMEG_0835), catalase/peroxidase (MSMEG_3461), alkyl hydroperoxidase (MSMEG_4890), alkyl hydroperoxide reductase (MSMEG_4891), thiol peroxidase MSMEG-3479), antioxidant (MSMEG_4753), and aldo/keto reductase (MSMEG_6746). Along these lines, enzymes responsible for synthesis of mycothiol (“unique” MSMEG_5129 and MSMEG_5261) in mycobacteria were found in the “dormant proteome.” Mycothiol is the functional equivalent of glutathione in mycobacteria (Newton and Fahey, 2002) and is associated with the protection of *Mtb* from toxic oxidants and antibiotics (Buchmeier et al., 2003). Mycothiol-dependent enzymes participate in detoxification of electrophilic components and inactivation of reactive oxygen and nitrogen species, reductions, and isomerizations (Newton et al., 2008). One of these reactions is the participation of mycothiol in formaldehyde oxidation performed by mycothiol-dependent formaldehyde dehydrogenase (MSMEG_4340, “unique”; **Supplementary Table S2**). Therefore, we estimated the level of thiols in *Msm* cells. The thiol concentration in dormant cells was 2.5 times higher than that in active cells (**Table 2**).

The proteome of dormant *Msm* contains highly represented enzymes responsible for detoxification of methylglyoxal and other reactive aldehydes: aldehyde dehydrogenase (MSMEG_2597) and a glyoxalase family protein (MSMEG_5680, “unique”), as well as enzymes inactivating active nitrogen compounds [alkylhydroperoxide reductase (MSMEG_4891), dihydrolipoamide dehydrogenase (MSMEG_0903), dihydro lipoamide succinyltransferase (MSMEG_4283), and alkyl hydroperoxidase (MSMEG_4890)]. The dormant proteome also contains an enzyme responsible for detoxification of oxidized lipids (glutathione S-transferase; MSMEG_5695, “unique”; **Supplementary Table S2**).

### Chaperones

In addition to the well-known chaperones that are well represented in both cell types, GroL (MSMEG_0880), DnaK (MSMEG_0709) and trigger factor MSMEG_4674, we found a “unique” chaperone for dormant cells: ClpB (MSMEG_0732). A significant increase in the expression of alpha-crystalline HspX (MSMEG_3932) was also found in dormant cells.

### Biosynthetic Processes

In comparison with proteome of active cells, the dormant proteome is characterized by the absence of several enzymes responsible for the biosynthesis of some important biomolecules: pyrimidines (MSMEG_3046), purines (MSMEG_3785, MSMEG_1647), pyridoxine (MSMEG_2937), thymidylates (MSMEG_2765), histidine (MSMEG_3209), cobalamin (MSMEG_6277), and pantothenate (MSMEG_6097).

At the same time, enzymes involved in cell wall synthesis were found in dormant cells. The most represented were MSMEG_2659, MSMEG_4231 (“unique”; **Supplementary Table S2**) and MSMEG_4229 (“unique”; **Supplementary Table S2**), which are involved in the synthesis of peptidoglycan and may be responsible for cell wall modification upon the transition to dormancy (Kudykina et al., 2011).

Dormant cells contain “unique” enzymes responsible for porphyrin synthesis (MSMEG_0953, MSMEG_0956, and MSMEG_2780), leucine synthesis (MSMEG_6271 and MSMEG_2379) and arginine synthesis (MSMEG_3769, MSMEG_3770, MSMEG_3773, and MSMEG_3774).

Dormant cells evidently accumulate storage materials: we found several enzymes responsible for the synthesis and accumulation of glycogen (MSMEG_4918), trehalose (MSMEG_6514 and MSMEG_6515 “unique”; **Supplementary Table S2**) and polyphosphates (MSMEG_2391). All these enzymes were found in the dormant cell proteome only (“unique” proteins).

### Degradation Processes

The proteome of dormant *Msm* is enriched for several enzymes that participate in the degradation of major cellular constituents, such as lipids (in addition to lipid hydrolysing enzymes found in active cells, there are additional “unique” enzymes in the dormant cell proteome: MSMEG_1821, MSMEG_1813, MSMEG_6008, MSMEG_6391, MSMEG_2938, MSMEG_5184, and MSMEG_6511). Similarly, enzymes with proteolytic activity were found in both types of cells. However, proteolytic enzymes are more diverse and more represented in the dormant cell proteome. Some of them are “unique” (MSMEG_0234, MSMEG_4200, MSMEG_4690, MSMEG_4672, MSMEG_3895, MSMEG_4673, MSMEG_2092, and MSMEG_0732 “unique”; **Supplementary Table S2**)

The expression of polynucleotide phosphorylase (MSMEG_2656) in the “dormant proteome” was observed. Polynucleotide phosphorylase is a component of RNA degradosomes involved in mRNA degradation (Py et al., 1996).

### Transport Across the Membrane

Active cells revealed 43 proteins that participate in the transport of different molecules across the plasma membrane, in contrast to dormant cells, which contained only nine proteins with the same function. Some of the “dormant” transport proteins were more abundant than in the active cell proteome [extracellular solute-binding protein (MSMEG_0643), oligopeptide transport (MSMEG_0639), ABC transporter (MSMEG_1954), “unique” and porin (MSMEG_5483)].

## Discussion

Comparison of protein profiles of active and dormant *Msm* cells revealed significant differences in protein abundance. Thus, 180 proteins (51%) were found to be represented in dormant cells and absent in active cells (“unique” proteins; **Supplementary Table S2**). At the same time, active cell proteome contained 193 proteins (53%) that were absent in the dormant cell proteome. This result differs significantly from published data obtained in other studies employing different models of mycobacteria dormancy. The number of differently expressed proteins found in the current study is much higher than that in such models as the “non-replicative anaerobic state” for *Mtb* (16–21 proteins) (Rosenkrands et al., 2002; Starck et al., 2004), the starvation model for *Mtb* (seven proteins) (Betts et al., 2002), and the late stationary phase (10 proteins) (Ang et al., 2014) estimated by 2D electrophoresis. This discrepancy could be explained by the fact that dormant cells in the present model, in addition to being in non-replicative state, possess properties (see section “Results”) that reflect the deeper state of dormancy (Young et al., 2005) of such cells in comparison with other published dormancy models.

We found only a few proteins that were accumulated in our dormant cells and in those of published models. In particular, α-crystalline homolog (heat shock protein HspX (MSMEG_3932) was detected in significant amounts in dormant *Msm* cells as well as in non-replicated, anaerobic *Mtb* (Wayne model) (Rosenkrands et al., 2002; Starck et al., 2004), *Msm* (Mishra and Sarkar, 2015), and *Mycobacterium bovis* (Boon et al., 2001). Evidently, this protein, which has chaperonin activity, is expressed under any stressful condition, such as starvation (Mishra and Sarkar, 2015), excess of iron in growth medium (Wong et al., 1999), heat or cold shock (Yuan et al., 1996).

Alanine dehydrogenase (MSMEG_2659) is also a well-known protein, the expression of which increases in published mycobacterial dormancy models in *Msm* (Hutter and Dick, 1998) and in *Mtb* (Jungblut et al., 1999; Rosenkrands et al., 2002). This protein participates in maintenance of NAD concentration when the final electron acceptor is limited (Hutter and Dick, 1998). In addition, we found heparin-binding hemagglutinin (MSMEG_0919) as a “unique” protein in dormant cells. By proteomic profiling using 2D electrophoresis, this protein was found to be more abundant in starved cells in Loebel dormancy model in *Mtb* (Albrethsen et al., 2013). The previous finding shows that heparin-binding hemagglutinin can be used as a marker for LTB infection (Hougardy et al., 2007).

In the Wayne dormancy model for *Mtb*, significant increase in protein expression took place among 48 proteins belonging to Dos regulon (Park et al., 2003; Schubert et al., 2015). The dormancy survival regulon, regulated by response regulator DosR appears to be essential for hypoxic survival in many mycobacterial species, including *M. tuberculosis* (Sherman et al., 2001; Voskuil et al., 2004; Leistikow et al., 2010), *M. bovis* BCG (Boon and Dick, 2002), and *Msm* (O’Toole et al., 2003). In our study we did not find DosR orthologs (MSMEG_3944/MSMEG_5244) in dormant cells and very few number of proteins potentially included in Dos regulon according to homological similarity with *Mtb* (MSMEG_2031, MSMEG_2032, MSMEG_0082, and MSMEG_3131) were found to be significantly expressed in dormant cells. We were not surprised by this result because, in the in long-lasting Wayne anaerobic model for *Mtb* (which is closer to the one in our study), proteins of the Dos regulon are much less expressed in comparison with the short-term non-replicative model (Rustad et al., 2008) and the same situation for vitamin C induced dormancy model, when DosR regulon was even downregulated (Albeldas et al., 2013).

**FIGURE 5:**
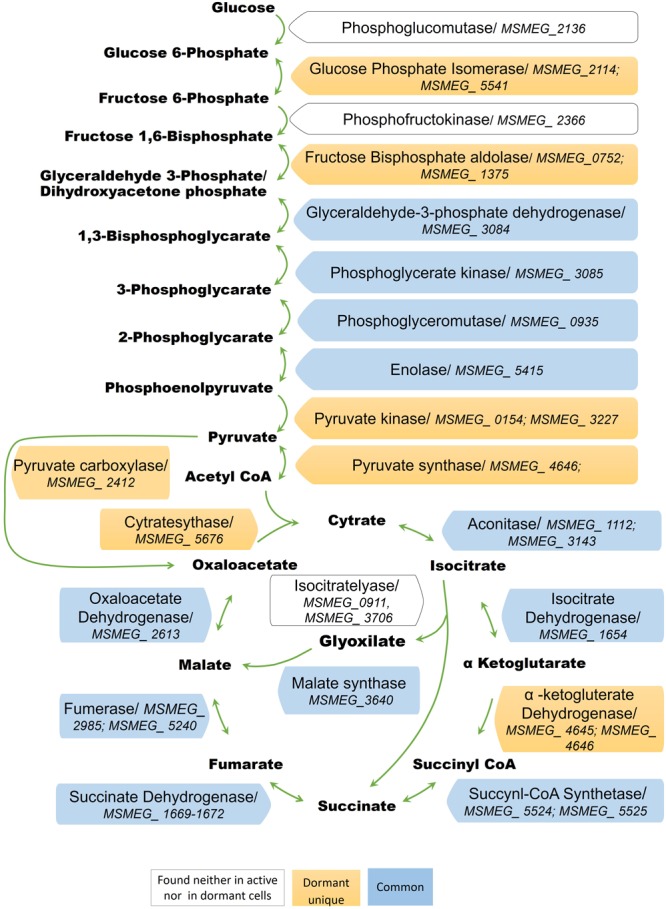
Enzymes of the glycolytic pathway, citrate cycle, and the glyoxylate shunt in the proteome of active and dormant *M. smegmatis.*

A visible feature of the “dormant proteome” is the high representation of enzymes involved in glycolysis (**Supplementary Table S1**). For *Msm* grown in Sauton medium, the carbon source in the dormant phase could be glycerol present in the medium, even after 2 months of storage (unpublished result). Although eight enzymes of the pathway from glycerol to pyruvate are well represented in the dormant cell proteome, one enzyme – glycerol-3-phosphate dehydrogenase (MSMEG_6761) – is absent, in contrast to the active cell proteome. This was confirmed by the very low level of this activity in the cell extract of dormant cells in contrast to active cells (**Table 2**) that makes conversion of glycerol an unlikely process. As an alternative, glucose could be converted to pyruvate by nine enzymes (**Figure 5**). We found that two enzymes of this pathway are “unique” to dormant cells (pyruvate kinase, MSMEG_3527; glucose phosphate isomerase, MSMEG_5541). The most represented enzyme is pyruvate kinase, which converts phosphoenolpyruvate to pyruvate. We confirmed the activity of some enzymes experimentally and found comparable specific activities of 3-phospo-glyceratekinase, glyceraldehyde-3-P-dehydrogenase, and pyruvate kinase in active and dormant cells (**Table 2**). These results make the conversion of glucose to pyruvate in dormant cells a distinct possibility. Because growth medium does not contain glucose, we could suggest that glucose-1-phosphate can be formed from glucan by α-glucan phosphorylase (MSMEG_4915), which is “unique” (**Supplementary Table S2**) or from glycogen by glycogen debranching enzyme (MSMEG_3186) and alpha-glucan phosphorylase (MSMEG_4915).

Another possibility is the hydrolysis of trehalose to glucose by trehalase. The accumulation of trehalose and the role of trehalase activity in maintaining the viability of dormant *Msm* cells during prolonged storage were found and discussed previously (Shleeva et al., 2017). Free glucose can be phosphorylated by glucokinase (MSMEG_1363) or by polyphosphate glucokinase (MSMEG_2760) to glucose 6-phosphate. The significance of glucose metabolism to *Mtb* persistence in mice was found (Marrero et al., 2013).

The fate of pyruvate, which accumulated in significant amounts in dormant *Msm* cells (Nikitushkin, personal communication), is not clear. Pyruvate could be converted to acetyl-coA by pyruvate synthase (MSMEG_4646) (“unique”; **Supplementary Table S2**), the reaction that plays role in maintaining cellular redox status. Pyruvate could also be converted to oxaloacetate by pyruvate carboxylase (MSMEG_2412) (“unique”; **Supplementary Table S2**). Could pyruvate be transformed to the end products of glycolysis like in anaerobic bacteria? To produce ethanol from pyruvate, the cell should contain pyruvate decarboxylase (conversion of pyruvate to acetaldehyde). Although this enzyme is not annotated in the *Msm* genome, there is another enzyme – indole-3-pyruvate decarboxylase (MSMEG_5735) – that is uniquely represented in the “dormant proteome” (“unique”; **Supplementary Table S2**). This enzyme can use pyruvate as a substrate along with its natural substrate indol-3-pyruvate (Schütz et al., 2003). The subsequent conversion of acetaldehyde to ethanol could be accomplished by ADH found in the “dormant proteome” (MSMEG_0127, “unique”; **Supplementary Table S2**, MSMEG_2079, MSMEG_6242). Activity of ADH was confirmed experimentally (**Table 2**).

Pyruvate could potentially be converted to lactate like in fermenting bacteria. However, fermentative lactate dehydrogenase (in contrast to FAD-dependent lactate dehydrogenase (MSMEG_2492) is not annotated in the *Msm* genome. LDH activity was not detected experimentally, however, NADH dependent reduction of pyruvate was observed (**Table 2**). In this context, extracellular accumulation of lactate by dormant *Msm* in significant amounts (Nikitushkin, personal communication, also see Zimmermann et al., 2015) remains an intriguing possibility. Thus, we cannot exclude the possibility that dormant bacteria can ferment during long storage using the glycolytic pathway. This would produce ATP under conditions of a non-functional respiratory chain.

The finding that all enzymes of the citric cycle are represented in the “dormant proteome” indicates the potential activity of this cycle under dormancy. However, under conditions where the electron transport pathway is inhibited (**Table 1**), the whole cycle cannot function. Zimmerman et al. (2015) suggested that under anaerobic conditions *Msm* may use the reductive branch of the Krebs cycle from pyruvate to succinate via malate and fumarate (reverse direction) with extracellular accumulation of succinate. This makes it possible to oxidize reduced equivalents formed in the glycolysis pathway (Zimmermann et al., 2015). As suggested, succinate efflux could be electrogenic and produce membrane potential (Zimmermann et al., 2015). It could be vital for ATP synthesis and maintenance of long-term cell survival. We cannot exclude a similar situation in our case, although the dormant cells in our experiments were not anaerobic (cells did not reduce methylene blue).

We found significant accumulation of the protein MSMEG_6227 (“unique”; **Supplementary Table S2**), that contains DNA-binding domain. According to the preliminary annotation, this protein is considered as a member of the PadR family of similar proteins with transcriptional regulatory activity. In particular, PadR negatively regulates padA (phenolic acid decarboxylase) in *Pediococcus pentosaceus* under the influence of toxic phenolic acids (Barthelmebs et al., 2000). Involvement of PadR-like proteins in multidrug resistance, virulence and cellular response to heating was established (Tran et al., 2008; Madoori et al., 2009). These proteins could also be important players in the maintenance of the bacterial dormant state. Firstly, PadR plays a role in transcriptional regulation of universal stress protein (Usp) (Gury et al., 2009), which can bind to ATP or intracellular oxygen and subsequently inhibit metabolism globally. Secondly, PadR regulates the transcription of genes coding for the synthesis of ABC transporters responsible for exporting antibiotics from cells (Lubelski et al., 2006; Madoori et al., 2009). This process could be responsible for the observed resistance of dormant mycobacterial cells to antibiotics (Shleeva et al., 2011; Salina et al., 2014). Because PadR accumulates in significant amounts in dormant cells, we cannot exclude its role in modification of DNA topology, similar to the compaction of DNA in dormant *Msm* cells caused by histone-like Hlp protein (Anuchin et al., 2010). Such structural changes could result in a global (unspecific) repression of transcription, which is a characteristic event upon transition from the active state to dormancy (Salina et al., 2009).

Dormant cells revealed accumulation of the transcription elongation factor NusA, which was not detected in active cell proteome (“unique”; **Supplementary Table S2**). *Escherichia coli* NusA stimulates RNA polymerase termination and pausing (Yakhnin et al., 2008). NusA can also act as a protector against protein aggregation under heat stress conditions (Li et al., 2013).

Found in dormant cells only, protein MSMEG_3335 (“unique”; **Supplementary Table S2**) belongs to IclR family and may play a role as a transcriptional repressor under dormancy. Thus, iclR (Icl regulator) is a repressor of the glyoxylate bypass operon in *E. coli* (Molina-Henares et al., 2006). Along these lines, we found no activity of the central enzyme of this shunt –Icl (**Table 2**), which shows a remarkable difference between dormant ovoid cells and non-replicative anaerobic *Msm* (Zimmermann et al., 2015) and *Mtb* cells in the Wayne model (Schubert et al., 2015).

We found SigB as a “unique” protein in the “dormant proteome” (“unique”; **Supplementary Table S2**), which plays a crucial function in promoting *Staphylococcus aureus* intracellular persistence. (Tuchscherr et al., 2015). This protein is a key regulator in the general stress response in Gram-positive bacteria (van Schaik and Abee, 2005) and contributes to resistance to both oxidative stress and carbon starvation in *Listeria monocytogenes* (Ferreira et al., 2001). In addition, we found MprA (MSMEG_5488) (“unique”; **Supplementary Table S2**), part of the stress-responsive two-component system MprAB that regulates the *in vivo* expression of sigB in *M. tuberculosis* (He et al., 2006).

In general, we found significantly fewer transcriptional regulators in the “dormant proteome” in comparison with the “active proteome.” Most regulators detected in dormant cells are repressors, which probably reflects global repression of metabolism upon transition to dormancy.

A remarkable feature of dormant cells is the presence of several different defense systems (destroying reactive oxygen and nitrogen species, aldehyde inactivation, detoxification of oxidized lipids). Evidently, these reactions make dormant cells less sensitive to stressful conditions in the absence of the main biosynthetic processes for substitution of damaged polymers.

Similarly, the finding of enzymes responsible for porphyrin synthesis (“unique”; **Supplementary Table S2**) in dormant cells is in line with the previously found substantial accumulation of copro- and uroporphyrins in dormant *Msm* cells (Nikitushkin et al., 2016). Observations indicate that the antioxidant activities of porphyrins act to protect bacteria (Patel and Day, 1999), animal tissues (Antonova et al., 2010), and animal mitochondria (Castello et al., 2008) against reactive oxygen species and toxic nucleophiles (Fuhrhop, 1974). It is highly probable that porphyrins may play a protective role and ensure the stability of dormant cells against unfavorable conditions or destructive factors.

Another feature of the “dormant proteome” is its enrichment in degradative enzymes, including proteases and lipases. On the one hand, these processes could eliminate damaged molecules in the absence of *de novo* synthesis or products of such degradation could be reutilized by the cell during prolonged storage, providing long-lasting “catabolic survival” of dormant cells.

In summary, we demonstrate, for the first time, the stability of proteins in dormant mycobacteria after prolonged storage and preservation of enzymatic activity of selected enzymes. Evidently, this stability is provided by high representation of different defense systems found in dormant cells and by cell structural modifications. We suggest that all proteins found in dormant cells could be divided into three groups: (1) those which are expressed during the transition from the active to the dormant state and further preserved in the dormant state; (2) those which are stored in dormant cells for further cell reactivation; (3) those which are functional and could play a role in maintaining cell metabolism, albeit at very low rate. We should stress that the ratio NADH/NAD is almost identical in active and dormant bacteria (although the concentration of dinucleotides in the dormant form was ∼10 times less than in active cells (**Table 2**). This means that reactions resulting in NADH generation took place within dormant cells; otherwise, the NADH concentration would quickly decrease due to the action of NADH-consuming enzymes like NADH-oxidase (**Table 2**). Detectable amounts of ATP and cAMP (**Table 1**) in dormant *Msm* cells also favors the maintenance of some level of metabolism under dormancy. At the same time, major biosynthetic processes, such as protein or RNA synthesis, are not active in dormant cells according to the low bacteriocidic activity of the corresponding antibiotics targeting these cells (**Table 1**). In general, this study provides a clue as to which biochemical processes could be active under dormancy to ensure long-term viability of dormant mycobacteria. This knowledge can be an important step in design of substances directed against dormant mycobacteria in order to combat latent TB. However, more studies should be undertaken for a detailed characterization of “dormant metabolism” under long-term, non-dividing conditions for *Mtb*.

## Author Contributions

AK and KT conceived and designed the experiments, analyzed the data, and wrote the manuscript. MS, KT, GD, and VN performed the experiments. KT prepared figures and graphs. All the authors read and approved the final manuscript.

## Conflict of Interest Statement

The authors declare that the research was conducted in the absence of any commercial or financial relationships that could be construed as a potential conflict of interest.

## Footnotes

1 https://www.alphalyse.com/wp-content/uploads/2015/09/Silver-staining-protocol.pdf

2 http://svitsrv8.epfl.ch/mycobrowser/smegmalist.html

## Supplementary Material

FIGURE S1 2D electrophoresis of different fractions obtained from active and dormant *M. Smegmatis* cells. **(A,C,E)** Active, early stationary phase cells; **(B,D,F**) Dormant cells after 1 month storage at room temperature. **(A,B)** Membrane fraction extracted by CHAPS. **(C,D)** Membrane fraction extracted by SDS. **(E,F)** Cytosol fraction. Each gel was stained by Coomassie followed by silver staining.

TABLE S1 The proteins found in cytosol and membrane fractions of active and dormant *M. smegmatis* cells. Active cells were harvested from early stationary phase; dormant cells were obtained after gradual acidification in stationary phase followed by 1 month storage at room temperature. 2D electrophoresis and protein analysis for cytosol and membrane (CHAPS and SDS extracts) were performed as described in M&M. Density estimation of each protein spot was performed in two technical replicates of the pooled samples obtained from four independent biological replicates for active and dormant bacteria. The average results are shown. The relative error for density values of each spot did not exceed 5%. In the columns marked as (^∗^), proteins were ranged according to their spot density from highest (1) to lowest (154 for active and 151 for dormant cells) representation in the proteome. Proteins were virtually absent in the proteome marked as “200.” If a protein with particular accession number is found in several spots, the corresponding rank was assigned for a spot with maximum density. If one spot contained several different proteins, the overall spot density was distributed proportionally between proteins. The proteins with the score three times less than the protein with maximum score in the spot were not ranked and marked as ND.

TABLE S2 The proteins found in cytosol and membrane (CHAPS and SDS extracts) fractions of dormant *M. smegmatis* cells and virtually absent in active cells (“unique” proteins). For details, see legend for **Supplementary Table S1**.
