# Supplementary figures and images for: Protein Composition of Mycobacterium smegmatis Differs Significantly Between Active Cells and Dormant Cells With Ovoid Morphology
Source: Front Microbiol. 2018 Sep 4;9:2083. doi: 10.3389/fmicb.2018.02083 (PMC6131537; doi:10.3389/fmicb.2018.02083)

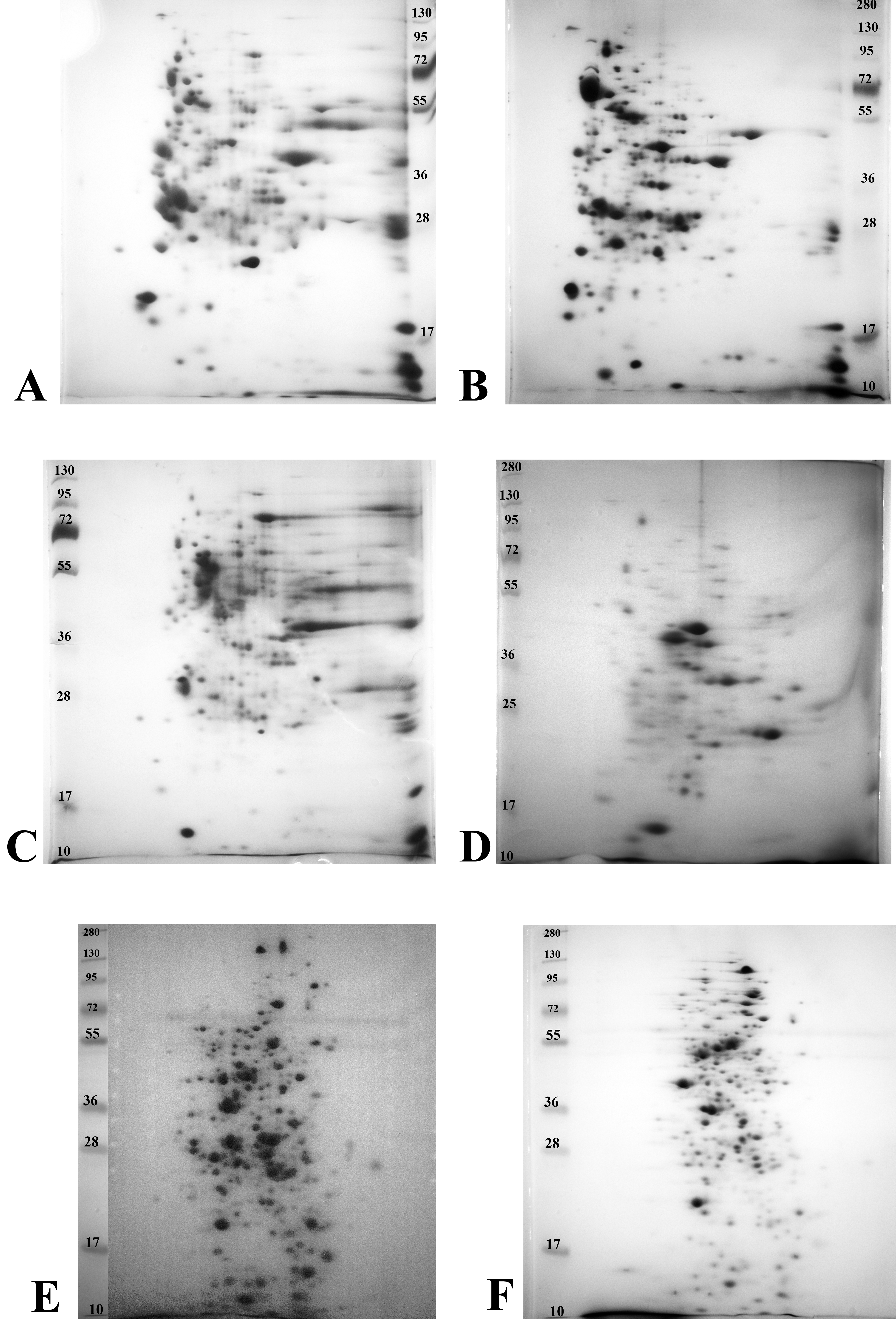

Supplement: FIGURE S1 — 2D electrophoresis of different fractions obtained from active and dormant M. Smegmatis cells. (A,C,E) Active, early stationary phase cells; (B,D,F) Dormant cells after 1 month storage at room temperature. (A,B) Membrane fraction extracted by CHAPS. (C,D) Membrane fraction extracted by SDS. (E,F) Cytosol fraction. Each gel was stained by Coomassie followed by silver staining. [file Image_1.TIF]
